# Preservation of 5300 year old red blood cells in the Iceman

**DOI:** 10.1098/rsif.2012.0174

**Published:** 2012-05-02

**Authors:** Marek Janko, Robert W. Stark, Albert Zink

**Affiliations:** 1 Department of Earth and Environmental Sciences, Ludwig-Maximilians-Universität München, Theresienstraße 41, 80333 Munich, Germany; 2 Center for NanoSciences, Ludwig-Maximilians-Universität München, Schellingstrasse 4, 80799 Munich, Germany; 3 FB Material- und Geowissenschaften, TU Darmstadt, Petersenstrasse 32, 64287 Darmstadt, Germany; 4 Center of Smart Interfaces, TU Darmstadt, Petersenstrasse 32, 64287 Darmstadt, Germany; 5 European Academy of Bolzano, Institute for Mummies and the Iceman, Viale Druso 1, 39100 Bolzano, Italy

**Keywords:** ancient erythrocytes, haemoglobin, protein degradation, Iceman, atomic force microscope, Raman spectroscopy

## Abstract

Changes in elasticity and structures of red blood cells (RBCs) are important indicators of disease, and this makes them interesting for medical studies. In forensics, blood analyses represent a crucial part of crime scene investigations. For these reasons, the recovery and analysis of blood cells from ancient tissues is of major interest. In this study, we show that RBCs were preserved in Iceman tissue samples for more than 5000 years. The morphological and molecular composition of the blood corpuscle is verified by atomic force microscope and Raman spectroscopy measurements. The cell size and shape approximated those of healthy, dried, recent RBCs. Raman spectra of the ancient corpuscle revealed bands that are characteristic of haemoglobin. Additional vibrational modes typical for other proteinaceous fragments, possibly fibrin, suggested the formation of a blood clot. The band intensities, however, were approximately an order of magnitude weaker than those of recent RBCs. This fact points to a decrease in the RBC-specific metalloprotein haemoglobin and, thus, to a degradation of the cells. Together, the results show the preservation of RBCs in the 5000 year old mummy tissue and give the first insights into their degradation.

## 1. Introduction

Examining mummies with sensitive analytic tools enables the reconstruction of their ancestry and genetic relationships [1,2], diet, diseases [2], living conditions, state of preservation and the mummification processes [3]. While many studies provided molecular evidence for the presence of infectious diseases in ancient populations, leading to deep insights into the evolution of such diseases [4,5], only a few reports on the recovery of blood from mummified bodies are available. Previous investigations, based on optical or electron microscopy data, postulated that blood remains or fragments could be preserved in mummies as old as 2000 years [6–10]. Although molecular verification of blood findings was not performed, detection of blood components was of major interest because it could give new perspectives on the lives and fates of our ancestors. Blood can indicate the general health status of an individual and it can be analysed to detect pathological conditions or to provide valuable information in forensic crime scene investigations.

One of the oldest forensic puzzles encompasses the death of the Tyrolean Iceman. This wet-mummy, commonly known as ‘Ötzi’, was presumably killed by an arrow. The corpse was found *ca* 5300 years later [11] in 1991 [12]. The mummy was exceptionally well preserved, and it still had intact connective tissue [13,14] and nervous system components [13]. However, in contrast to the good overall preservation of its tissue, no blood has been found so far. Thus, it was initially assumed that the blood had disintegrated owing to autolysis within the corpse [13]. Later, X-ray and computed tomography images of the Iceman body gave the first hints of blood residues. A prehistoric arrowhead that was surrounded by inhomogeneous soft-tissue areas was located between the rib cage and the left scapula. The areas were interpreted as being dehydrated haematomas [15,16] and associated with a lesion in the left subclavian artery that could have led to haemorrhagic shock and the Iceman's death [17]. Haemoglobin, a common blood protein, was detected in a skin wound on the Iceman's right hand using a guaiac-based test. However, it did not provide evidence for intact blood cells [18]. Recently, a microscopic analysis of immunochemically stained histological tissue samples indicated the possible presence of blood residues [19].

Here, we report the direct detection of red blood cells (RBCs) in tissue samples from the Iceman with an atomic force microscope (AFM) and Raman spectroscopy. Single and clustered RBCs were found, and their characteristic Raman spectra were obtained. The spectra contained Raman bands of proteinaceous remnants, most likely fibrin, which indicates the formation of a blood clot. The Raman spectra, however, also document a degradation of the cells. Their spectral intensity was approximately an order of magnitude weaker than that of recent RBCs. Additional elasticity measurements on the cells imply a loss in RBC stability—which also points to degradation.

## 2. Material and methods

Iceman tissue was obtained by punch biopsies from the stab trauma to the right hand (sample A) [18] and from the wound under the left spina scapulae on the Iceman's back (sample B) [15]. The extracted tissue was rehydrated for 48 h in a 9.5 parts formaldehyde (2%) and 0.5 parts Brij 35 solution, and subsequently fixed with 4 per cent formaldehyde (formalin) for 2 h, dehydrated in an ascending alcohol series and embedded into paraffin wax. Histological specimens were obtained by cutting 2–4 µm thick transverse sections and transferring them onto glass slides. Before AFM analysis, the paraffin was dissolved in xylene. Finally, the sections were rehydrated with a descending alcohol series, rinsed with ultrapure water and dried under ambient conditions [14]. As a reference, a recent human tissue sample, taken from a volunteer and processed in the same manner as the Iceman samples, was used. Additionally, fresh capillary whole blood was drawn from the fingertip of a volunteer, applied to a glass slide and left to dry for 6 h. Furthermore, a glass slide was coated with a meshwork of fibrin, an essential protein formed during the blood clotting process. The preparation of fibrin was carried out following the protocol of Riedel *et al.* [20].

Particles with the approximate size and shape of RBCs were identified with an inverted optical microscope (Axiovert 135; Zeiss, Oberkochen, Germany). Then, high-resolution images were taken with a NanoWizard-II AFM (JPK Instruments, Berlin, Germany). The AFM was operated in the intermittent contact mode. Silicon cantilevers (BS Tap 300; Budget Sensors, Redding, CA, USA) with nominal spring constants of 40 N m^–1^, resonance frequencies of 300 kHz and tip radii of 10 nm were used.

Additionally, within the fixed samples, individual putative RBCs were analysed by AFM nanoindentation so as to assess elasticity. Force curves of recent (*n* = 363) and mummified (*n* = 213) samples were obtained by indenting an AFM tip with a defined radius of 300 nm (LRCH 250; Team Nanotec, Villingen-Schwenningen, Germany) into the corpuscle surface. The nominal spring constant, *k*_c_, of the cantilever was 40 N m^–1^, and the loading force for each measurement was limited to 500 nN. Four recent RBCs and two corpuscles extracted from the arrowhead entry wound on the back of the mummy (sample B) were tested. Only (putative) RBCs that were lying flat on the glass substrate were analysed to ensure good mechanical contact with the substrate. The numerical value for Young's modulus *E* was obtained from fitting a Hertzian model [21] on the force curves. Sneddon's extension [22,23] of the Hertzian model was used to calculate the deformation *δ* of the flat elastic sample surface penetrated by a rigid spherical indenter (AFM tip) of radius *R.* The spherical indenter geometry was assumed because the indentation depth of the AFM tip into the sample was small compared with the tip radius. The samples' Young's modulus was calculated from2.1
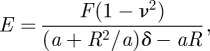
with the sample deformation given by2.2
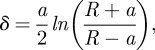
with *F* the applied force, *a* the radius of the contact area between the tip and the sample, and *ν* the Poisson ratio of the material analysed. The Poisson ratio was set to 0.5, assuming an incompressible material.

For the molecular analysis, a confocal Raman spectroscope (WITec alpha 300 R; WITec GmbH, Ulm, Germany; excitation wavelength 532 nm) was used. To avoid photodegradation, laser power was limited to 1.0 mW. The spectrometer was operated with an 1800 g mm^–1^ grating. The spectral resolution was 1 cm^−1^ per CCD-pixel. Three different positions were analysed for each sample, and at least three single spectra, with 180 s of integration time, were taken at each position. Owing to the confocal set-up of the microscope, Raman spectra were collected from a sample area with 300 nm diameter and a focal depth of approximately 1 µm.

## 3. Results

**Figure 1.**
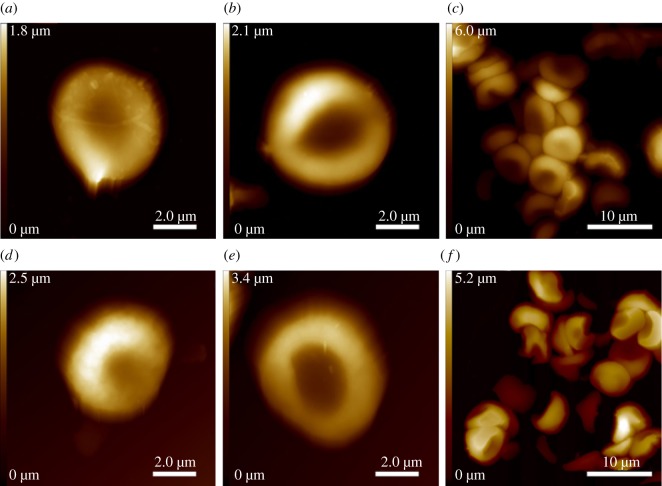
AFM images of RBCs. (*a*,*b*) Single RBCs from recent human tissue. (*c*) An assembly of RBCs. (*d*,*e*) Single corpuscles found in Iceman sample A and sample B are shown. An assembly of several randomly distributed corpuscles, similar to those found within the recent sample (*c*), are displayed in image (*f*). The imaged corpuscles (*d*–*f*) feature the characteristic discoid and concave surface of RBCs.

One corpuscle with a structure likely to be a RBC [24] was found in the hand wound tissue of the Iceman (figure 1*d*), and two single corpuscles were detected within the arrowhead wound sample. Sample B furthermore showed an agglomeration of several randomly distributed particles (figure 1*f*). The selected corpuscles exhibit a discoidal, concave surface with a diameter between 5.8 and 6.4 µm (figure 1*d*,*e*). The concave shape is typical for RBCs and arises during the early stages of development in the bone marrow when the cell nuclei are discarded, leaving behind an impression on the membrane. The dip in the membrane of the corpuscle was 0.7–1.1 µm deep. For comparison, RBCs from a recent human tissue sample, similar in structure and appearance to those found in the Iceman samples, are shown in figure 1*a*–*c*. The mean cell diameter of the recent RBCs was 6.3 ± 0.4 µm, which matches the average diameter of the ancient corpuscle. The cells are also similar when comparing the mean area and volume of the RBCs (table 1). The measured height of the recent cells and of the mummy particles differ more; however, the variation is still within the error margin.

**Table 1.** Dimensions of recent RBCs and ancient corpuscles.

sample	quantity	height (µm)	diameter (µm)	area (µm^2^)	volume (fl)
recent RBCs	19	2.0 ± 0.5	6.3 ± 0.4	31.0 ± 3.7	40.0 ± 12.2
ancient corpuscle	3	2.5 ± 0.2	6.0 ± 0.3	28.8 ± 3.2	42.1 ± 4.1

**Figure 2.**
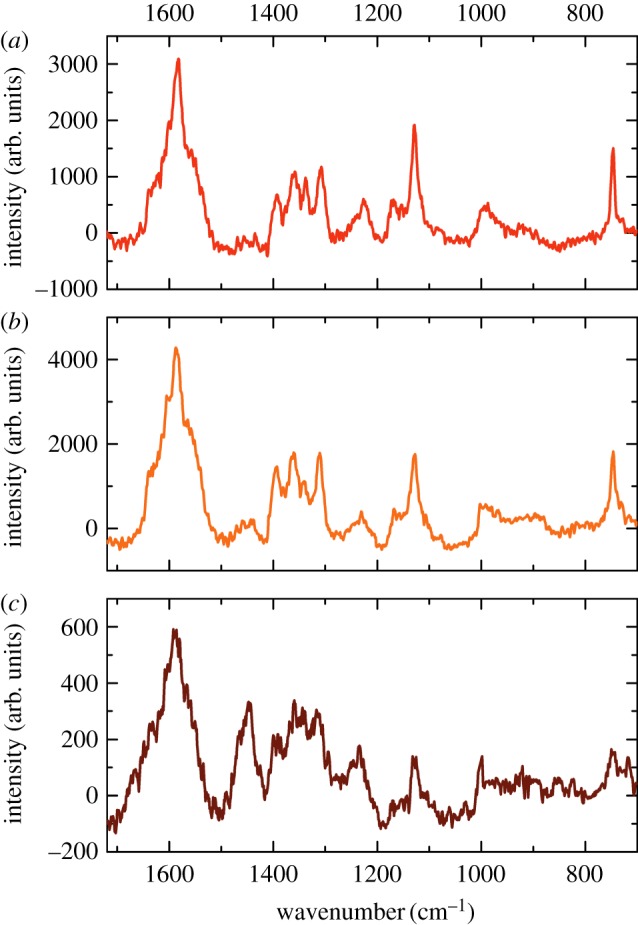
Raman spectra of air-dried whole blood (*a*), a single red blood cell (*b*), and the corpuscle found in the Iceman tissue sample A (*c*). All spectra show peaks at 1586, 1395, 1308 and 747 cm^−1^, which are characteristic of porphyrin. Apart from some bands, the spectra show considerable similarities.

To assess the chemical composition and structural conformation of the ancient corpuscles, Raman measurements were performed. The spectral fingerprint region of the particle in Iceman sample A was compared with that of an air-dried recent whole blood sample and with that of recent single RBCs. The recent single RBCs were subjected to the same sample preparation as that of the mummy tissue before examination. All Raman spectra shown in figure 2 exhibit distinct bands at 1586, 1395, 1308 and 747 cm^−1^, which are assigned to the stretching vibration modes ν_37_, ν_20_/ν_29_, ν_21_ and ν_15_ of porphyrin. Porphyrin is the characteristic building unit of the major RBC protein haemoglobin [25–27]. Furthermore, bands that are typical for other protein components, such as the twisting deformation mode of methylene at approximately 1230 cm^−1^, were present. The spectrum of Iceman sample A additionally shows two small peaks at approximately 1665 and 1248 cm^−1^ and two prominent bands at 1446 and 1002 cm^−1^. The latter are assigned to the proteinaceous deformation vibration of methyl δ(CH_3_) and methylene δ(CH_2_) molecules and to the vibration mode of phenylalanine [14,28]. The two small peaks at 1665 and 1248 cm^−1^ are assigned to the amide I (C=O stretching) and the amide III (C−N stretching and N−H in plane deformation modes) groups [29]. A detailed band assignment is shown in table 2. Comparing the intensities of the spectra, a strong decrease in scattering efficiency can be observed for the ancient particle. The intensity of the Raman spectrum of Iceman sample A is approximately an order of magnitude weaker than that of the recent blood samples, although the ratio between the band intensities within the spectrum remained largely unchanged.

**Table 2.** Raman peak assignment of Iceman sample A.

whole blood wavenumber (cm^−1^)	red blood cell wavenumber (cm^−1^)	Iceman sample A wavenumber (cm^−1^)	assignment	mode	literature
		1665	amide I	ν(CO)	[30]
1636	1639	1633	ν_10_	ν(C_α_C_m_)_as_	[25]
1584	1586	1588	ν_37_	ν(C_α_C_m_)_as_	[26,27]
1562	1563	1565	ν_2_	ν(C_β_C_β_)	[25,26]
—	—	1512	ν_38_		[26]
—	—	1491	ν_3_	ν(C_α_C_m_)_sym_	[25]
1468	—	—			
1453	1459	—		CH_2_/CH_3_	[27]
1434	1441	1446		δ(=C_b_H_2_)_sym_	[25–27]
1392	1395	1397	ν_20_, ν_29_	ν(pyr quarter-ring)	[25]
1361	1362	1360			
1338	1340	1343	ν_41_	ν(pyr half-ring)_sym_ δ(=C_b_ H_2_)_sym_	[25–27]
1308	1312	1314	ν_21_	δ(C_m_H)	[25,27]
1274	1275	1279			
—	—	1248	amide III	ν(CN)	[29,31]
1225	1229	1233		prop δ(CH_2_) twisting	[25]
1170	1166	—	ν_30_	ν(pyr half-ring)_as_	[25,26]
—	—	1157			
1150	—	—	ν_14_	ν(C_β_C_1_)_sym_	[25,26]
1129	1129	1129			
1083	1079	—		δ(=C_b_H_2_)_as_	[25]
—	—	1063			
991	997	1002	ν_45_	ν(C_β_C_1_)_as_	[28]
—	—	973		ν(Cc–Cd)	[25]
924	—	926		γ(=C_b_H_2_)_sym_	[26]
—	896	901			
—	—	823	γ_10_	γ(C_m_H)	[28]
747	746	747	ν_15_	ν(pyr breathing)	[25,26]

**Figure 3.**
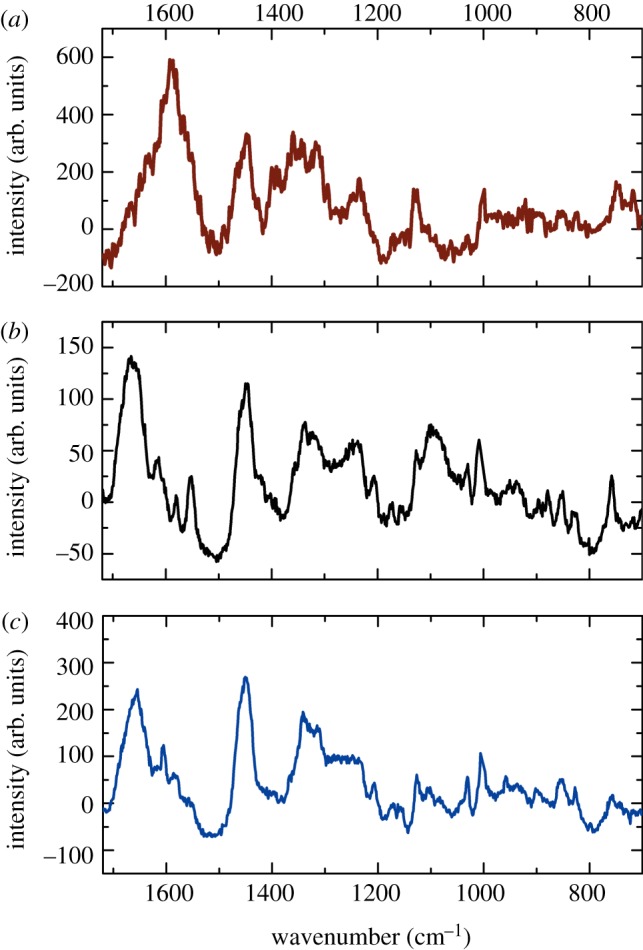
Raman spectra of the corpuscle in Iceman sample A (*a*), a fibrin meshwork (*b*), and the corpuscles found in the Iceman tissue sample B (*c*). The spectrum obtained from sample B strongly differs from that of sample A. It has features with considerable similarities to the spectrum of fibrin.

**Figure 4.**
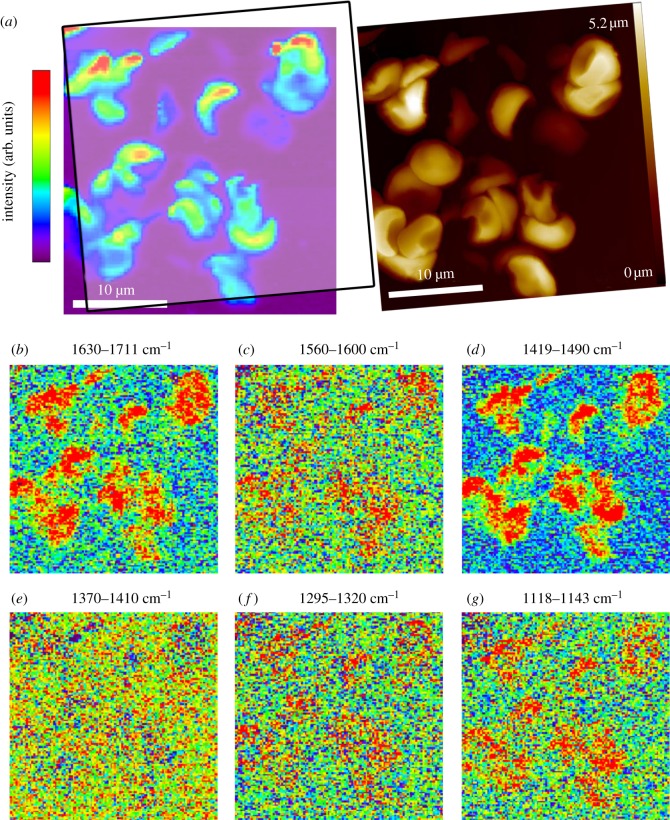
Raman scan and AFM image (30 × 30 µm) of the agglomerated corpuscle in Iceman sample B. The scan in (*a*) represents the intensity distribution of the Rayleigh scattered light. For comparison the corresponding AFM topography image is shown. (*b*–*g*) The datasets of the Raman scan filtered for porphyrin or protein-specific Raman bands around 1656, 1586, 1449, 1395, 1308 and 1125 cm^−1^ wavenumbers. Red indicates regions with strong Raman intensity, and blue indicates low Raman intensities. The examined molecule vibrations largely occurred at the positions of the ancient particles.

When the fingerprint region from 700 to 1720 cm^−1^ in the particles found in Iceman sample B was analysed*,* hardly any Raman bands indicated the presence of RBCs (figure 3). A characteristic spectrum of the corpuscles in sample B is shown in figure 3*c*. The first strong Raman band observed is the amide I peak at approximately 1656 cm^−1^, corresponding to the C=O stretching vibration ν(CO) typical for proteins. Further prominent bands appear between 1620 and 1600 cm^−1^, originating from the C=C vibrational stretch mode ν(CC) of the amino acids tyrosine or phenylalanine. Similar to Iceman sample A the corpuscles in sample B feature strong methyl δ(CH_3_) and methylene δ(CH_2_) bands at approximately 1449 cm^−1^ and a distinct peak at 1002 cm^−1^, which represents the vibration mode of phenylalanine. There were also bands assigned to the deformation of methylene δ(CH_2_) molecules between 1380 and 1300 cm^−1^ [30,31]. Finally, the peak appearing at approximately 758 cm^−1^ can be attributed to the aromatic ring breathing of tryptophan [30]. As shown in figure 3, the Raman bands observed in Iceman sample B (figure 3*c*) exhibit similarities to those observed when examining pure fibrin (figure 3*b*). The intensities of the spectra were largely similar; however, the amount of sample material analysed has to be taken into account. The reference fibrin spectrum was obtained from a thin fibrin layer a few hundred nanometres thick, and the average particle spectrum shown in figure 3*c* was recorded on a 2 µm thick particle. Table 3 also indicates that Raman bands from sample B imply the presence of fibrin instead of RBCs. Nonetheless, some correlations between porphyrin vibration modes and the location of the ancient particles can be drawn from two-dimensional Raman scans. The data displayed in figure 4*a* associate the colour-coded Rayleigh scattering intensity of a Raman scan to an AFM topography image. Figure 4*b*–g shows the Raman data filtered for selected molecule vibrations. Red indicates sample regions with strong Raman scattering. Areas with low intensity are in blue. Specific molecule vibrations are predominant in areas that correlate with the position of the ancient particles. Figure 4*c*,*e*,*f* illustrates the intensity distribution around the bands at 1586, 1395 and 1308 cm^−1^, which are associated with the porphyrin vibration modes ν_37_, ν_20_/ν_29_ and ν_21_. In the wavenumber range 1370–1410 cm^−1^, no Raman scattering occurred (figure 4*e*). The molecule vibrations ν_37_ and ν_21_, however, show a moderate signal at the position of the ancient particles. The highest Raman signals are observed in the wavenumber range of the protein-specific bands, e.g. the amide I peak around 1656 cm^−1^ (figure 4*b*), the methyl δ(CH_3_) and methylene δ(CH_2_) band at approximately 1449 cm^−1^ (figure 4*d*) and the C–N stretch mode around 1125 cm^−1^ (figure 4*g*).

**Table 3.** Raman peaks assigned for Iceman sample B.

RBC wavenumber (cm^−1^)	fibrin wavenumber (cm^−1^)	Iceman sample B wavenumber (cm^−1^)	assignment	mode	literature
	1666	1656	amide I	*ν*(CO)	[30]
1639			*ν*_10_	*ν*(C_α_C_m_)_as_	[25]
	1617	1605		*ν*(C=C) Phe, Tyr	[29,30]
1586			*ν*_37_	*ν*(C_α_C_m_)_as_	[26,27]
	1581	1583		*ν*(CCH) Pro, Hypro	[29]
1563			*ν*_2_	*ν*(C_β_C_β_)	[25,26]
1441	1447	1449		*δ*(CH_3_, CH_2_)	[29]
	1402	1399		*δ*(CH_2_)	[31]
1395			*ν*_20_, *ν*_29_	*ν*(pyr quarter-ring)	[25]
1340	1339	1340	*ν*_41_	*ν*(pyr half-ring)_sym_ *δ*(=C_b_H_2_)_sym_	[25–27]
1312	1317	1315	*ν*_21_	*δ*(C_m_H), *δ*(CH_2_)	[25,27,31]
—	1252	1248	amide III	*ν*(CN)	[29,31]
1229				prop *δ*(CH_2_) twisting	[25]
	1208	1208		*ω* (CH_2_)	[31]
	1174	1173		C–H bend Tyr	[30]
1166			*ν*_30_	*ν*(pyr half-ring)_as_	[25,26]
—	1156	1157		C–C/C–N str	[30]
1129	1125	1125		C–N str	[30]
—	1101	1102			
1079	1075	1084		*δ*(=C_b_H_2_)_as_	[25]
—	1031	1032		C–H in-plane Phe	[30]
997	1004	1002	*ν*_45_	*ν*(C_β_C_1_)_as_, *ν*(CC) aromatic ring Phe	[28,30,31]
					
—	956	957		*ν*(Cc–Cd)	[25]
—	937			*γ*(=C_b_H_2_)_sym_	[26]
896	896	898			
—	854	855	*γ*_10_	*γ*(C_m_H), *ν*(CC) aromatic	
				ring Tyr	[28,30]
	829	827		*ν*(CC) aromatic ring Tyr	[30]
	758	758		aromatic ring breath	[30]
746			*ν*_15_	*ν*(pyr breathing)	[25,26]

**Figure 5.**
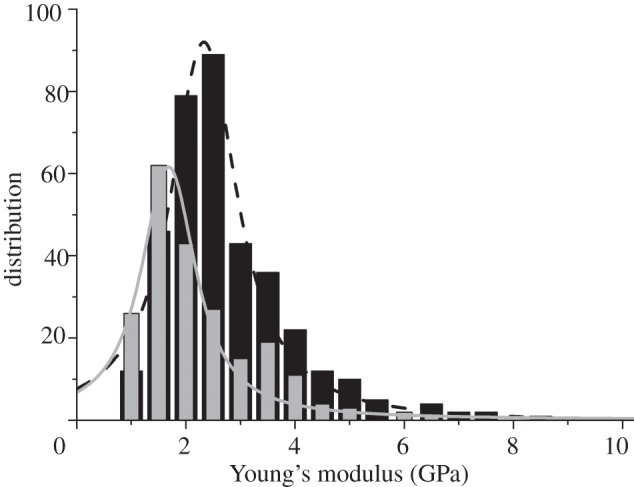
The distribution of Young's moduli from the corpuscle of Iceman sample B and contemporary single RBCs. Young's modulus for the mummy particles (grey) is significantly lower than Young's modulus for the recent RBCs (black).

An analysis of the mechanical properties of RBCs can give further insights into their structural integrity. In the circulatory and cardiovascular system, they are subject to deformation owing to varying flow conditions. To withstand wear, the cells feature an elastic discoidal membrane. This structure, constituting a minimum energy configuration [32], and the dynamic remodelling of their spectrin cytoskeleton [33], enables them to pass through thin capillaries and reach tissues. Because changes in the mechanical properties of RBCs are an indicator of disease and can provide an insight into their molecular preservation, nanoindentation measurements were carried out to assess the elasticity of the fixed, ancient corpuscles. A histogram with Young's modulus of the mummy corpuscles (grey) and the reference RBCs (black) is shown in figure 5. By way of illustration, a Lorentzian distribution is additionally fitted. For the fixed mummy samples, a mean Young's modulus of 2.0 ± 1.0 GPa with a distribution maximum of 1.7 GPa was determined. The measurements conducted on fixed, recent RBCs yielded a mean value of 2.5 ± 1.2 GPa, and the distribution maximum was 2.3 GPa. The difference between the mean Young's moduli is statistically significant and was analysed with the independent two-sample Student's *t*-test.

## 4. Discussion

To study the presence of human RBCs in the tissue of the 5300 year old Iceman, tissue samples extracted from two Iceman wounds were examined. Performing AFM measurements, isolated single corpuscles with the approximate size and shape of normal RBCs were identified in Iceman samples A and B. The corpuscles featured a discoidal and concave shape, which is typical for RBCs, and their morphology did not demonstrate any evidence of degradation, damage or disorder. Moreover, the dimensions of these ancient corpuscles matched those of the similarly prepared reference RBCs. Additionally, a cluster of several randomly agglomerated particles was revealed in sample B. In this sample, all particles greater than approximately 5 µm showed artificial interfaces, which are presumably cutting edges that arose from the preparation of the histological specimens, i.e. slicing of the tissue with a microtome. Apart from these sectioned areas, the particles showed typical RBC morphology. These first results indicate that RBCs have been preserved for more than 5000 years in the wound tissue of the mummy.

To further confirm the presence of RBCs, Raman spectra were taken from the Iceman samples and compared with reference whole blood and reference RBC spectra. The stretching vibration modes ν_37_, ν_20_/ν_29_, ν_21_ and ν_15_, which are characteristic of haemoglobin [25–27], dominated the spectrum of the corpuscle in Iceman sample A. This suggested that the ancient particle is a RBC. However, the scattering intensity of the ancient spectrum was approximately an order of magnitude weaker than that of the recent blood samples. The intensity *I* of a Raman band depends on4.1
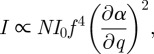
where *N* is the number of scattering molecules within a sample, *I*_0_ is the intensity of the excitation laser, *∂*α*/∂q* is the change in polarizability of the exited molecules and *f* is the frequency of the excitation laser. Because the intensity and the frequency of the laser were kept constant during the measurements, and because the bands in the ancient particle spectrum can be clearly assigned to molecule vibrations with defined polarizability, the change in the scattering intensity was most probably caused by the reduced number of scattering molecules within the ancient particle. This fact indicates decomposition of the Iceman RBC, in association with the degradation of the majority of the RBC-specific haem compounds and hence the reduction in scattering molecules. RBC degradation is mainly caused by the action of reactive oxygen species (ROS) such as superoxide 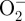 radicals that are released during the autoxidation of oxygen-loaded haemoglobin (oxyHb) [34]. During the dismutation of superoxide, hydrogen peroxide (H_2_O_2_) can also be generated [35]. Both ROS cause oxidative stress within the RBC, which eventually leads to the decomposition of proteins due to fragmentation of their peptide chains. The effectiveness of ROS is governed by the oxyHb autoxidation rate of approximately 0.5–3% per day [35,36] and the vast amount of molecular oxygen that can be bound within a single RBC. Besides the action of ROS, damage of the RBC can also be induced by freezing and thawing, as observed in cryopreservation procedures. Thereby RBC injury can be attributed to events such as intracellular and extracellular ice formation, excessive cell shrinkage, osmotic stress or dehydration [37]. Such mechanisms affect the RBC on the macroscopic scale but have less influence on the microscopic scale, i.e. the protein content and the molecular composition.

The ancient RBC spectrum also showed bands at 1665, 1446, 1248 and 1002 cm^−1^. These were assigned to the amide I band typical for proteins, the deformation vibration of methyl δ(CH_3_) and methylene δ(CH_2_), the amide III band that represents peptide bonds and the vibration mode of phenylalanine. All these bands are common in proteins such as collagen or fibrin [30,31,38,39], and the amide III band is also occasionally observed in the spectra of air-dried RBCs and met-RBCs, as discussed by Asghari-Khiavi *et al.* [25]. Compared with reference protein spectra and those of the particle found in Iceman sample B (figure 3), the protein band at 1665 cm^−1^ is very faint in the ancient RBC spectrum, whereas the other protein bands are more pronounced. Thus, the Raman spectrum of Iceman sample A is a complex composition of haemoglobin and other protein spectra.

In summary, the altered spectral signature and intensity in comparison with data of recent RBCs were most probably caused by a modification of the haemoglobin and, consequently, the porphyrin. The proteinaceous compounds may have been less strongly affected. Some molecule vibrations could have also been impaired by the effect of blood clotting and the formation of proteins such as fibrin.

Changes in molecular structure owing to protein compounds are even more evident in the corpuscle from Iceman sample B. Although the corpuscle morphology resembles the structure of regular RBCs, the Raman spectra significantly differed from the reference RBC spectra. The corpuscles mostly lacked the previously mentioned porphyrin modes and predominantly showed bands characteristic of fibrin or other proteins such as collagen (see the electronic supplementary material). Fibrin is the end product of a complex cascade of coagulation reactions that are initiated at the moment of vascular injury. In the first step, platelets become activated and then become adherent to the damaged vessel wall. They form a primary haemostatic plug. Meanwhile, the enzyme thrombin is produced. Thrombin then cleaves fibrinogen and catalyses the polymerization of fibrin, which subsequently generates a meshwork around the platelet plug and reinforces it [20,40]. The Raman spectra and AFM images thus indicate that the single particles and the agglomerate of corpuscles in the tissue of sample B are the remnants of a haemostatic plug that formed around the Iceman's arrowhead wound. Nonetheless, it is surprising that, although the Raman spectra of the Iceman's RBCs are dominated by bands characteristic for fibrin, neither a meshwork nor single fibrin fibrils were detected in our AFM images. We therefore infer that the fibrin surrounding the haemostatic clot decomposed over time, leaving behind only RBCs and possibly some fibrin fragments.

Although our results show more complex RBC spectra, they are in agreement with measurements on recent dried human blood spots conducted by Virkler *et al.* [41], who showed that dried whole blood is chemically heterogeneous, and its Raman spectrum could be a linear combination composed mainly of the two Raman active components: haemoglobin and fibrin. The clotting process that occurs while blood dries explains the spectral combination. One hypothesis was that fibrin was formed during the clotting, and that it was therefore found in a large concentration in the dried blood spots.

Finally, when analysing the Raman spectra of the pre-processed tissue, the effect of the sample preparation must be taken into account. The ancient and the recent RBC samples were processed into histological sections, i.e. the tissue was fixed with formaldehyde, embedded into paraffin and cut with a microtome. The paraffin was then dissolved with xylene, and the sample was subsequently rehydrated in a descending alcohol series. The dehydration effects of alcohol were found to cause degradation of RBCs because it weakens the membrane–water interactions of the cell membrane [42]. This can lead to the loss of some cellular components and can therefore cause changes in the Raman spectra. Dehydration and the effect of fixatives, such as formaldehyde and glutaraldehyde, on RBC Raman spectra were also reported by Asghari-Khiavi *et al.* [25]. In their study, they showed that the Raman spectra of RBCs, which were fixed in a formaldehyde–glutaraldehyde mixture and embedded into paraffin, were very similar to the spectra of air-dried RBCs. This indicates that the processing of dried RBCs into histological tissue sections does not cause additional alterations of the RBC Raman spectra. Furthermore, because the reference RBCs and Iceman samples were processed equally, preparation-induced differences are rather unlikely. Therefore, we conclude that the processing of the tissue had little influence on the comparative spectroscopic examination.

Deformation and failure phenomena of hierarchical protein materials are observed in physiologically extreme conditions and in the progression of disease [43]. The structural proteins and thus the shape, molecular structure and the elasticity of RBCs are also prone to disease-specific alterations [44]. Reduced mechanical deformability, together with increased RBC membrane stiffness, are reported in infection with the malaria parasite [45–47]. A similar phenotype appears in blood disorders such as sickle-cell disease, hereditary elliptocytosis or hereditary spherocytosis, in which the deformability of the RBC membrane is reduced and the cell shape is strongly altered [47–49]. Owing to the pre-processing of the tissue samples, however, we cannot draw conclusions on any disease-specific mechanical changes of the RBCs. Nevertheless, a relative comparison between equally processed recent and mummy RBCs helps us to assess the degree of tissue preservation. AFM nanoindentation measurements revealed changes in the mechanical behaviour of the RBCs. Young's modulus of the ancient RBCs in sample B was 2.0 ± 1.0 GPa, whereas the modulus of the equally processed recent RBCs averaged 2.5 ± 1.2 GPa.

Cross-linking owing to fixation with formaldehyde [50], age [51] or disease-specific influences would lead to an increased membrane stiffness of RBCs and, thus, to an increase in Young's modulus. Our measurements on ancient RBCs, however, show a decrease in Young's modulus associated with a lower stiffness of the ancient RBCs. Together with the reduced Raman scattering intensity, the softening indicates a degradation of the RBCs. Possible degradation processes include scaffold damage due to crystallization of ice during freezing, irradiation with UV light or wound healing-specific transformation processes that occur during the stages of blood clot degradation [52].

The fragmentation of the RBC cytoskeleton proteins such as the spectrin and actin filaments will cause a destabilization of the cell membrane. In addition to the aforementioned mechanisms, cleavage of membrane proteins and degradation of the cytoskeleton by the fragmentation of their protein peptide chains can also be induced by the action of ROS. The various degradation processes would ultimately lead to the softening of the RBC membrane.

The elasticity values determined are given for fixed recent and ancient RBCs, which were prepared following the same protocol. Obviously, the preparation comprises the mechanical properties of a tissue by the formation of methylene bridges that cross-link polypeptide chains. Thus, no conclusions on health status can be drawn from the elasticity measurements. However, comparing the elasticity values of both specimens, it is clear that the ancient samples were softer. This is in line with the observation that less Raman scattering occurred in the ancient RBCs. Both effects can be explained by a degradation of the proteinaceous content of the RBCs.

In summary, the morphology and the Raman fingerprint of some corpuscles point to remnants of a haemostatic clot. This observation confirms that the Iceman sustained several injuries before his death. AFM imaging revealed RBCs with normal morphology. Blood disorders caused by RBC membrane defects, such as sickle-cell disease, elliptocytosis or spherocytosis, can thus be excluded. Nanoindentation measurements show that the elasticity of the ancient RBCs is slightly reduced, which suggests that they suffered from degradation. Complementary Raman spectroscopy also indicates a degradation of the blood cells. Nevertheless, our examinations show an unambiguous identification of RBCs in a 5300 year old mummy.
